# Cost‐Consequence Analysis of a Dementia Specialist Training Program for Home Care Workers: Results From a Stepped‐Wedge Cluster‐Randomised Controlled Trial

**DOI:** 10.1111/ajag.70239

**Published:** 2026-10-05

**Authors:** Yared Belete Belay, Anita Goh, Briony Dow, Christa Dang, Steven Savvas, Frances Batchelor, Lidia Engel

**Affiliations:** ^1^ Health Economics Group, School of Public Health and Preventive Medicine Monash University Melbourne Victoria Australia; ^2^ The University of Melbourne Melbourne Victoria Australia; ^3^ National Ageing Research Institute Melbourne Victoria Australia; ^4^ Edith Cowan University Perth Western Australia Australia; ^5^ Deakin University Melbourne Victoria Australia

**Keywords:** cost‐effectiveness, dementia, health outcomes, homecare workers, informal care

## Abstract

**Objectives:**

This paper reports the health economic component of the broader Promoting Independence Through Quality Dementia Care at Home (PITCH). The broader PITCH trial primarily evaluated the effect of dementia training on home care workers' competence in providing dementia care; this analysis evaluated intervention costs and selected secondary client and caregiver outcomes.

**Methods:**

The PITCH intervention was tested via a multi‐site national pragmatic stepped‐wedge cluster randomised clinical trial, conducted across three Australian states. A micro‐costing approach was used to estimate intervention and resource use costs. Secondary outcomes for clients and caregivers, including quality of life (QoL), caregiver burden and dyadic interaction, were assessed. Means (SDs) described costs and outcomes, with linear and multilevel mixed‐effects regression used to estimate crude and adjusted mean differences, respectively.

**Results:**

There were no significant improvements in most client and caregiver health, QoL, caregiver burden or dyadic interaction outcomes during the intervention phase, with decreases in proxy‐reported Dementia QoL Utility (DEMQOL‐U) and client self‐reported visual analogue scale (EQ VAS). Improvements in caregiver burden and dyadic interaction were observed but were not statistically significant. Total costs increased from AU$68,925 in the control to AU$133,660 in the intervention phase, with an adjusted mean difference of AU$67,146 (95% CI: AU$16,957 to AU$117,334).

**Conclusions:**

This health economic analysis should be interpreted separately from and alongside, the main PITCH trial findings on the primary outcome of home care workers' competence. Within this secondary analysis, PITCH was associated with higher total costs during the intervention phase and no clear evidence of improvement in the selected secondary client and caregiver outcomes.

## Impact Statements

**Policy Impact:**

This national trial provides important evidence on the costs and consequences of specialist dementia workforce training. While client outcomes were unchanged, the programme strengthened home care workers' competence and reduced informal care costs. Policymakers should prioritise efficient, scalable delivery models and evaluate longer‐term system impacts before large‐scale investment.

**Practice Impact:**

PITCH enhanced home care workers' knowledge, attitudes and perceived competence in dementia care, reinforcing the importance of workforce capability in home‐based services. Providers should optimise training format, participant numbers and engagement strategies to improve efficiency and maximise potential downstream benefits for clients and caregivers.

## 1 Introduction

The increase in the older adult population in Australia is associated with a growing prevalence of chronic illnesses and disabilities, resulting in an increased demand for aged care services [1]. In Australia, formal aged care services are provided either within residential aged care facilities or through home care services—community‐based services at individuals' own homes [2]. Residential aged care is an option for individuals who can no longer live at home or need ongoing help with daily activities and health care [3]. Home care workers (HCWs), also known as community aged care support workers, provide general household assistance, rather than residential aged care services [2]. HCWs working in the aged care sector are responsible for providing assistance to older individuals, ranging from personal hygiene and domestic tasks to social support and healthcare aid, with the aim of maintaining their clients' independent living in the community [1, 2]. In Australia, a Certificate III in Individual Support is the minimum entry‐level qualification for aged care roles, including home care workers, while staff working in residential aged care may require additional training in medication assistance, infection prevention and control and dementia care [4].

Dementia is one of the main causes of disability and dependence in older people [5]. Dementia entails a progressive decline in cognitive function and memory, adversely affecting daily activities [6]. This irreversible condition significantly impacts the person with dementia's health outcomes. While there is currently no cure for dementia, existing care focuses on improving the overall well‐being and maintaining the quality of life (QoL) of the person with dementia [7]. People with dementia often prefer to reside in their own home while receiving care and support [8]. Tailoring aged care services to the unique situation of people with dementia is paramount. A client‐centred care approach fosters not only improvements in quality of care but also results in positive outcomes for the care recipients [9]. Therefore, it is important for HCWs and family caregivers to have competence and awareness of the specific needs and services for individuals with dementia in a home setting [10].

To date, interventions supporting HCWs in providing care to people with dementia have received little attention. Ensuring that HCWs have sufficient confidence and knowledge in providing care to people with dementia is expected to improve the QoL of clients and reduce burden/strain to caregiver. However, there is currently a lack of evidence on the most effective delivery mode for dementia training programmes for HCWs, and many programmes appear unfeasible with poor adherence [11]; however, such programmes are being developed and evaluated (e.g., NIDUS‐professional [12]), and the evidence base is growing. There is also no existing evidence on the cost‐effectiveness of many of these programmes, with only one previous study examining the delivery cost of such a programme in the United Kingdom, which was estimated to be ₤6423 (₤137 per client), which is around AUD$12,189 (AUD$260 per client) [12].

This study evaluated the costs and consequences of the Promoting Independence Through Quality Dementia Care at Home (PITCH) programme, directed to HCWs, working with people living with dementia and their family caregivers. While the primary outcome was to enhance the competence of HCWs in dementia care, this economic evaluation focused on the trial's secondary outcomes that included health and QoL outcomes for the clients and their caregivers. Using a cost‐consequence analysis approach, the study examined the impact of the PITCH intervention on health outcomes and resource use by individuals with dementia and their caregivers from a limited societal perspective.

## 2 Methods

### 2.1 Study Design and Setting

The PITCH study was a multi‐site national pragmatic stepped‐wedge cluster randomised clinical trial conducted across three States of Australia: Victoria (VIC), New South Wales (NSW) and South Australia (SA). The trial involved 18 clusters consisting of seven centres (service providers). The protocol details have been previously published elsewhere [10]. This trial received approval from an appropriate institutional Human Research Ethics Committee. Written informed consent was obtained from all participants prior to their participation in the study.

### 2.2 Eligibility and Recruitment

This study involved individuals living with dementia, family caregivers and HCWs. The PITCH intervention was provided to HCWs aged 18 years and above involved in providing services to clients living with dementia receiving Australian government‐funded home care through either the Home Care Packages Program (HCP) or the Commonwealth Home Support Programme (CHSP). Adult clients diagnosed with any stage of dementia who were receiving home care service packages and had no plans of moving to aged care facilities within 6 months were recruited to participate, as were their family caregivers who provided more than 6 h of care a week. Home care providers identified potentially suitable clients and informed them about the study. If clients or their family carers agreed to be contacted, a member of the research team approached them to discuss the study, confirm eligibility and invite them to participate. Written informed consent was obtained from each participant (clients, family caregivers and HCWs) prior to participating in the PITCH study (For further details, see the published trial protocol) [10].

### 2.3 Intervention

The PITCH intervention is an educational and training programme. It was co‐designed with people with dementia and their caregivers [13]. The primary goal of the PITCH intervention is to enhance HCWs' competence in delivering care, thereby likely improving health and care outcomes for clients and their family caregivers as secondary outcomes. The training was originally developed as a face‐to‐face training package, but a shift to an online format was necessary in response to the COVID‐19 pandemic. The face‐to‐face education programme was adapted for live (synchronous) online delivery. The online programme consisted of three facilitator‐led 2‐h interactive workshop sessions for each cluster, replacing the original two half‐day face‐to‐face workshops. The educational content, learning objectives, facilitator and training materials remained identical to the face‐to‐face programme, with only the mode of delivery and session scheduling modified to facilitate remote participation [10]. In the control scenario of this stepped‐wedge trial, clusters begin without the PITCH training programme, maintaining usual practices until they transition to the intervention phase.

### 2.4 Outcomes

For the health economics analysis reported in this paper, the PITCH intervention secondary outcomes were evaluated, which included health and QoL outcomes for the clients and their caregivers. All outcomes were obtained at three time‐points: baseline, 6 and 12 months. Client QoL was evaluated using self‐reported and proxy (carer)‐reported values using the EQ‐5D‐5L (EuroQol 5 Dimensions 5 Levels) and DEMQOL (Dementia Quality of Life) [14, 15]. The EQ‐5D‐5L is a widely used tool for measuring health‐related QoL [16]. The Australian tariff was used to derive the health state utility value based on EQ‐5D‐5L descriptive system [17]. The visual analogue scale (EQ VAS), an integral aspect of EQ‐5D‐5L, provided a direct way for participants to rate their own perception of their health within the defined range of 0 to 100, with higher values indicating better health [18]. The DEMQOL (28 items) and DEMQOL‐proxy (31 items) ask respondents about their feelings, memory and everyday life in the last week, with response options ranging from a lot (Level 1) to not at all (Level 4) [19]. The DEMQOL responses were converted to utility values using a scoring algorithm based on the value generated from the UK general population [19]. Clients' social care‐related QoL was assessed using the Adult Social Care Outcomes Toolkit (ASCOT‐SCT4) [20, 21]. The ASCOT responses were converted to a scale of 0–1 (with scores anchored between 0 for being dead and 1 for the ideal state) using the scoring algorithm based on preferences of the UK general population [22].

The severity of dementia was assessed using the Rowland Universal Dementia Assessment Scale (RUDAS), which includes evaluations of memory, praxis, language, judgement, drawing and body orientation—a score below 23 out of a maximum of 30 indicates severe impairment [23]. The presence and severity of neuropsychiatric symptoms over the past month in clients and caregiver distress were reported by family caregivers using the Neuropsychiatric Inventory (NPI‐12) [24]. The daily functional activities of people with dementia living in the community were assessed through the Disability Assessment for Dementia Scale (DAD) [25]. Carer outcomes included the distress on the NPI‐12, QoL using the EQ‐5D‐5L and caregiver personal and role strain using the Zarit Burden Interview (ZBI) self‐report scale [26]. Additionally, the client‐carer relationship was examined using the Dyadic Relationship Scale (DRS), which was completed by the family caregiver [27].

### 2.5 Measurement and Valuation of Resource Use

#### 2.5.1 Cost of PITCH Intervention

The PITCH intervention was costed using a micro‐costing approach based on study records. The components of the intervention included: (i) the time of the trainer to prepare, travel and deliver the workshops; (ii) HCWs' time to travel and attend the workshops; (iii) travel cost; (iv) costs of workshop materials; (v) catering; and (vi) time of a research assistant to organise the workshops. Personnel time was costed at an hourly rate (trainer: AU$62, HCWs: AU$32.5, research assistant: AU$39) and included 30% on‐costs (such as superannuation, leave and other employee entitlements). The costing took into account the delivery mode, where six workshops were delivered face‐to‐face over 2 days (7 h in total) and 12 online workshops were delivered over 2 days (6 h in total). Two scenarios were costed: one assuming that service providers pay their HCWs to do the training using industry wages (which reflects the approach taken in this study), with a second scenario assuming that providers expect HCWs to do the training in their own time. Total costs were estimated as well as cost by mode of administration.

#### 2.5.2 Client Resource Use

Besides the cost of the intervention, we also measured clients' resource use during the duration of the study using the Resource Utilisation in Dementia Lite version (RUD Lite) [28]. This tool contains questions regarding health service use, including: (1) formal aspects such as health professional visits, medication usage, emergency department visits and hospital admissions; and (2) informal aspects such as care time and domestic assistance. Unit costs for professional visits (e.g., general practitioner/physician visit, neurologist visit) were obtained from the Australian Medicare Benefit Schedule (MBS) 2020 [29]. The unit costs of hospital admission and emergency visits were determined from the Australian National Hospital Cost Data Collection (NHCDC) for public hospitals, round 24 (Year 2019–2020) [30]. For further details on unit costs, see Tables S3 and S4. Costs for home care and domestic assistance were sourced from price quotes from the Home Care Package and the Commonwealth Home Support Program [31]. Additionally, we used the Australian Bureau of Statistics data to determine per‐hour payment or salary rates for professionals such as cleaners [32]. The costs of medication were estimated using data from the Australian Pharmaceutical Benefits Scheme (PBS) [33], relying on the Dispensed Price for Maximum Quantity.

This analysis employed a limited societal perspective and all resource use and outcomes were estimated for the duration of each phase (i.e., 6 months). Informal care time was measured by assessing the time dedicated by caregivers to support clients. Caregiver time was primarily allocated to three types of services: activities of daily living (ADL), instrumental activities of daily living (IADLs) and supervision [34]. Caregiving time was valued by multiplying the reported hours dedicated to each task by its respective hourly wage estimated, using the ‘replacement approach’ based on data from the Australian Bureau of statistics [32]. Hours dedicated towards ADL tasks were costed using the hourly wage of nursing support and personal care workers, while IADLs were estimated using hourly rates of bookkeepers, and supervision costs were based on housekeeper wages [35]. All costs are presented in 2020 Australian dollars ($), which is the reference year for the trial.

### 2.6 Statistical Analysis

The analysis followed the Consolidated Health Economic Evaluation Reporting Standards (see Table S5). Costs and outcomes were computed for each time period and compared to the usual care, where home care services were delivered by HCWs who had not yet received the PITCH training. Quality‐Adjusted Life Years (QALYs) were calculated using the area under the curve approach by multiplying the time between visits for each of the three periods by the respective utility score measured at the end of each visit. An intention‐to‐treat approach was taken. Missing data were imputed using the Multiple Imputation method with chained equations. Imputations were conducted using the Predictive Mean Matching technique in combination with K‐Nearest Neighbours imputation with a parameter value of 10. Imputation was conducted at the level of total costs for each cost category (e.g., health professional visit costs, hospital admission costs). A total of 70 imputation sets were generated, using cluster categories, participant sex, age, visit status and intervention status as predictor variables. Costs and outcomes were described using mean (SD). Crude mean differences were estimated and compared using linear regression. Multilevel mixed‐effects regression analysis was conducted to compare adjusted mean differences between control and intervention phases. This analytical approach employed a mixed‐effects regression model to adjust the crude mean, thereby accounting for both fixed effects (such as participant age, gender, intervention and visit) and random effects (participant cluster category). Baseline adjustments were not made in this study due to the stepped‐wedge design, where the intervention was introduced sequentially across different phases. A sensitivity analysis was conducted assuming that service providers anticipate HCWs to undertake the training in their personal time, resulting in an estimated cost of $218 per recipient for the intervention (rather than $908 per recipient used in the base case analysis when service providers were assumed to compensate HCWs at industry wages for training).

## 3 Results

### 3.1 Participant Characteristics and Baseline Outcomes

This study included 60 client and caregiver dyads recruited from 16 of the 18 PITCH trial clusters, as client–caregiver dyads were only recruited within these 16 clusters. At visit 2 (6 months), 32 dyads transitioned to the intervention phase but the study only had data from 28 clients. By Visit 3 (12 months), all remaining dyads (24) had shifted to the intervention phase (see Table S1). Fifty‐two per cent (52%) of clients were female, with a mean age of 83 years (SD = 13). Sixty‐seven per cent (67%) of caregivers were female with a mean age of 68 years (SD = 14) (Table 1).

**TABLE 1:** Demographic characteristics of the participants.

	Client *n* (%)	Family caregiver *n* (%)
Gender		
Male	29 (48)	20 (33)
Female	31 (52)	40 (67)
Age (mean [SD])	83 (13)	68 (14)
State		
VIC	19 (32)	19 (32)
NSW	9 (15)	9 (15)
SA	32 (53)	32 (53)
Education		
No formal schooling	1 (2)	—
Primary school	10 (21)	2 (4)
Secondary or high school	22 (46)	20 (42)
Trade community or TAFE college	13 (27)	6 (13)
Bachelors degree	1 (2)	11 (23)
Masters degree	1 (2)	3 (6)
PhD	—	1 (2)
Other	—	5 (10)
Formal dementia diagnosis		
Yes	43 (77)	N/A
No	11 (20)	N/A
Not sure	2 (3)	N/A
Type of dementia		
Alzheimer's disease	19 (53)	N/A
Vascular dementia	5 (14)	N/A
Mixed	3 (8)	N/A
Other types of dementia*	3 (8)	N/A
Unknown/not specified	6 (17)	N/A
Caregiver relationship to client		
Daughter	N/A	19 (32)
Wife	N/A	13 (22)
Husband	N/A	12 (20)
Son	N/A	7 (12)
Other	N/A	8 (13)
Shared household with client		
Yes	N/A	32 (53)
No	N/A	28 (47)
Level of informal care contribution		
1%–20%	N/A	8 (17)
21%–40%	N/A	7 (15)
41%–60%	N/A	6 (13)
61%–80%	N/A	6 (13)
81%–100%	N/A	20 (42)
Working status		
Yes	N/A	9 (18)
No	N/A	42 (82)
Income source		
Pension	21 (55)	40 (85)
Savings	10 (26)	5 (11)
Other**	7 (19)	2 (4)

Abbreviation: N/A, not applicable.

*Lewy body dementia, frontotemporal dementia, mild cognitive impairment, or mixed dementia.

**Government benefits or family assistance.

Baseline outcomes for clients and family caregivers are reported in Table 2. For client outcomes, the mean (SD) for the EQ‐5D‐5L utility, ASCOT, DEMQOL‐U and EQ VAS scores were 0.79 (SD = 0.31), 0.85 (SD = 0.15), 0.61 (SD = 0.15) and 78 (SD = 21), respectively. Proxy‐reported scores were lower compared to self‐reported client scores. Caregiver self‐reported utility scores on the EQ‐5D‐5L were 0.91 (SD = 0.08) and 82 (SD = 13.2) on the EQ VAS. Strain levels were measured using DRS strain, indicating that clients reported less strain (5.2, SD = 2.3) compared to family caregivers (8.7, SD = 3.9); clients also reported more positive aspects of care as measured by DRS positive (18, SD = 4.7) compared to family caregivers (16, SD = 4.4).

**TABLE 2:** Participants' characteristics and baseline outcome.

	Mean (SD)
Cient outcomes (self‐reported)	
RUDAS	18 (6.5)
EQ‐5D‐5L	0.79 (0.31)
DEMQOL‐U	0.61 (0.15)
ASCOT	0.85 (0.15)
EQ VAS	78 (21)
Client strain	
DRS positive	18 (4.7)
DRS strain	5.2 (2.3)
Client outcomes (proxy reported)	
EQ‐5D‐5L	0.61 (0.31)
DEMQOL‐U	0.59 (0.15)
EQ VAS	66 (19)
Disability measured by DAD	51 (31)
Neuropsychiatric symptoms (NPIQ)	
Severity	6.7 (6.6)
Distress	7.3 (9.3)
Carer outcomes (self‐reported)	
EQ‐5D‐5L	0.91 (0.08)
EQ VAS	82 (13.2)
Carer strain (carer‐reported)	
DRS positive	16 (4.4)
DRS strain	8.7 (3.9)
ZBI	28 (17)

Abbreviations: ASCOT, Adult Social Care Outcomes Toolkit; DAD, Disability Assessment for Dementia Scale; DEMQOL, Dementia Quality of Life; DRS, Dyadic Relationship Scale; EQ VAS, EuroQol Visual Analogue Scale; EQ‐5D, EuroQol 5 Dimension 5 Level; NPIQ, Neuropsychiatric Inventory; ZBI, Zarit Burden Interview.

Table 3 illustrates baseline costs (extrapolated for 6 months) across various healthcare categories. For the person living with dementia, hospital admission costs were the highest ($22,620, SD = 102,464), followed by other services ($7068, SD = 7544), health professional visit costs ($1662, SD = 2750), emergency visit costs ($411, SD = 2029) and medication costs ($2164, SD = 7599). In terms of informal care, care provided for ADLs incurred a mean cost of $9467 (SD = $12,541), IADLs had a mean cost of $10,536 (SD = $10,899) and supervision contributed to $6807 (SD = $10,511), resulting in the mean total informal care cost of $26,810 (SD = $25,988) per dyad. The mean total costs for formal and informal care yielded $60,735 (SD = $104,284) per dyad.

**TABLE 3:** Formal and informal caregiver costs over 6 months (at baseline).

Categories	Baseline
Formal healthcare services	Mean ± SD
Health professional visit costs	1662 (2750)
Hospital admission costs	22,620 (102,464)
Emergency department visit costs	411 (2029)
Total other services costs^a^	7068 (7544)
Medication costs	2164 (7599)
Total formal care cost	33,925 (106,641)
Informal care costs	
Activities of daily living	9467 (12,541)
Instrumental activities of daily living	10,536 (10,899)
Supervision	6807 (10,511)
Total informal care cost	26,810 (25,988)
Total (formal + informal)	60,735 (104, 284)

^a^ District nurse, home help/health assistant, meals on wheels, day care, transportation.

For Scenario 1 (representing the RCT methodology), the total cost of delivering the PITCH intervention to 18 clusters and 213 HCWs equated to the total cost of AU$54,467 (online: AU$21,997; face‐to‐face: AU$31,725). This represents a cost to each aged care provider of AU$3026 or AU$306 per HCW.

For Scenario 2, the costs equated to: total cost: AU$13,073 (online: AU$6556 or face‐to‐face: AU$6517), which represents a cost to the aged care provider of AU$726 or AU$73 per HCW. Further details can be found in Table S2.

For this analysis, the aggregate intervention costs were divided among the dyads (60 dyads) included in the intervention phases to determine the intervention costs per dyad. This resulted in a cost per dyad receiving intervention of AU$908 in Scenario 1 and AU$218 in Scenario 2.

### 3.2 Mixed‐Effects Regression Analysis

Table 4 presents the results of the multilevel analysis.

**TABLE 4:** Mean cost and outcomes during the intervention and control phases (Multilevel analysis).

Outcomes and costs	Crude mean (SE)		Crude mean difference (95% CI)	Adjusted mean difference (95% CI)^a^
	Control phases	Intervention phases		
Clients outcomes (self‐reported)				
EQ‐5D‐5L	0.76 (0.04)	0.71 (0.05)	−0.06 (−0.17,0.06)	−0.07 (−0.18, 0.05)
DEMQOL‐U	0.6 (0.02)	0.61 (0.02)	0.002 (−0.05,0.05)	−0.03 (−0.05,0.05)
ASCOT	0.85 (0.02)	0.79 (0.02)	−0.05 (−0.12, 0.01)	−0.08 (−0.18, 0.02)
EQ VAS	77 (2.4)	69 (3.1)	−7.8 (−16, −0.06)*	−14 (−28, −0.4)*
Client strain				
DRS positive	19 (0.55)	19 (0.61)	0.12 (−1.5, 1.7)	1.05 (−2.04,4.13)
DRS strain	5 (0.26)	5.4 (0.31)	0.42 (−0.4, 1.25)	0.85 (−0.73, 2.42)
Clients outcomes (proxy reported)				
EQ‐5D‐5L	0.6 (0.04)	0.55 (0.05)	−0.05 (−0.16, 0.07)	−0.05 (−0.16, 0.07)
DEMQOL‐U	0.59 (0.02)	0.54 (0.02)	−0.04 (−0.09, 0.002)	−0.04 (−0.09, −0.004)*
EQ VAS	65 (2.2)	63 (2.5)	−2.1 (−8.7, 4.5)	−2.3 (−8.72, 4.16)
Disability measured by DAD	50 (3.7)	43 (3.7)	−7.2 (−17.4,2.9)	−5.7 (−15, 4)
Neuropsychiatric symptoms (NPIQ)				
Severity	6.4 (0.74)	5.4 (0.61)	−1 (−2.8,0.86)	−1.03 (−2.80, 0.74)
Distress	7.1 (1)	6.1 (0.82)	−1 (−3.5, 1.6)	−0.82 (−3.28, 1.65)
Patient QALY				
QALY‐ self‐reported EQ‐5D‐5L	0.38 (0.02)	0.35 (0.02)	−0.03 (−0.09,0.03)	−0.03 (−0.09,0.02)
QALY‐ proxy‐reported EQ‐5D‐5L	0.3 (0.02)	0.28 (0.02)	−0.02 (−0.08,0.04)	−0.02 (−0.08,0.03)
QALY‐ self‐reported DEMQOL‐U	0.3 (0.01)	0.3 (0.01)	0.01 (−0.02,0.03)	−0.01 (−0.03, 0.02)
QALY‐proxy‐reported DEMQOL‐U	0.29 (0.01)	0.27 (0.01)	−0.02 (−0.04, −0.01)*	−0.02 (−0.04, −0.01)*
QALY‐ self‐reported ASCOT	0.42 (0.01)	0.4 (0.01)	−0.03 (−0.06, 0.01)	−0.03 (−0.06, 0.01)
Carer outcomes (self‐reported)				
EQ‐5D‐5L	0.91 (0.01)	0.89 (0.02)	−0.02 (−0.07,0.03)	−0.02 (−0.06, 0.03)
EQ VAS	82 (1.6)	81 (1.8)	−0.31 (−5.1,4.5)	−0.83 (−5.3, 3.6)
Carer strain (self‐reported)				
DRS positive	16 (0.51)	15 (0.5)	−1 (−2.4,0.37)	−0.71 (−2.04, 0.63)
DRS strain	8.5 (0.45)	9.3 (0.46)	0.8 (−0.5, 2.1)	0.65 (−0.59, 1.9)
ZBI	27 (2)	25 (1.8)	−2.6 (−7.8,2.6)	−3.66 (−8.6, 1.26)
Carer QALY				
QALY‐carer EQ‐5D‐5L	0.45 (0.01)	0.44 (0.01)	−0.01 (−0.03, 0.01)	−0.01 (−0.03, 0.01)
Cost				
Health professional visit costs	2174 (454)	3100 (524)	925 (−488,2339)	961 (−428, 2350)
Hospital admission costs	20,481 (11,473)	52,670 (18,549)	32,189 (−13,473, 77,852)	32,948 (−10,888, 76,784)
Emergency department visit costs	366 (217)	1450 (566)	1084 (−196, 2364)	1057 (−208, 2322)
Other services costs	18,910 (5419)	47,899 (9097)	28,989 (7916, 50,063)*	28,309 (9019, 47,599)*
Medication costs	1927 (782)	2684 (1150)	757 (−2055, 3568)	624 (−1883, 3,131)
Formal care cost (total)	43,858 (12,761)	107,803 (21,359)	63,945 (12,803, 115,087)*	62,791 (−12,803, 115,087)
Activities of daily living	8750 (1376)	9,696 (1,343)	945 (−2821, 4711)	526 (−3140, 4192)
Instrumental activities of daily living	9640 (1222)	9556 (1305)	−84 (−3548, 3380)	−650 (−4044, 2744)
Supervision	6677 (1252)	5697 (1223)	−980 (−4438, 2478)	−1302 (−4703, 2100)
Informal care costs (total)	25,067 (2945)	24,949 (3048)	−118 (−8466, 8230)	−1659 (−9868, 6,551)
Formal + Informal costs	68,925 (12,959)	132, 752 (21,188)	63,827 (12,259, 115,394)*	65,358 (15, 518, 115, 198)*
Intervention cost	0 (0)	908 (0)		
Total costs (including intervention cost)	68,925 (12,959)	133,660 (21,188)	64,735 (13,167, 116,302)*	67,146 (16,957, 117,334)*
Scenario analysis	68,925 (12,959)	132,970 (21,188)	64,044 (12,477, 115,612)*	66,456 (16,267, 116,644)*

^a^ Multilevel analysis, participant age, gender, intervention and visit as fixed effects, while cluster category as a random effect.

* Significant difference.

#### 3.2.1 Client Outcomes

*Client self‐reported* EQ‐5D‐5L utility scores were 0.76 (SE = 0.04) for the control phase and 0.71 (SE = 0.05) for the intervention phase, with a crude mean difference of −0.06 (95% CI: −0.17, 0.06). The adjusted mean difference was −0.07 (95% CI: −0.18, 0.05), indicating no significant difference between the control and intervention phases. Similar results were observed for other client outcome measures, including DEMQOL‐U, ASCOT and DRS scores. For EQ VAS, although client‐reported scores indicated significantly higher scores during the control phase than the intervention phase, when EQ VAS was proxy‐reported by the caregiver, the difference was no longer significant.

*Proxy‐reported* DEMQOL‐U indicated a significant decrease during the intervention phase (mean = 0.54, SE = 0.02) compared to the control phase (mean = 0.59, SE = 0.02), with an adjusted mean difference of −0.04 (95% CI: −0.09, −0.004). Neuropsychiatric symptoms measured by NPIQ, both severity and distress, exhibited a decrease during the intervention phase, with adjusted mean differences of −1.03 (95% CI: −2.80, 0.74) for severity and −0.82 (95% CI: −3.28, 1.65) for distress. Minor differences were observed in QALYs derived from either patient self‐reported or proxy‐reported EQ‐5D‐5L utility and DEMQOL‐U, with significance found only in QALYs based on proxy‐reported DEMQOL‐U.

#### 3.2.2 Caregiver Outcomes

No significant differences were observed between the control and intervention phases in both crude and adjusted mean differences for caregiver EQ‐5D‐5L, EQ VAS and DRS scores. ZBI scores revealed a decrease during the intervention phase (mean = 25, SE = 1.8) compared to the control phase (mean = 27, SE = 2), with an adjusted mean difference of −3.66 (95% CI: −8.6, 1.26) although this difference was not statistically significant.

In comparing costs between the control and intervention phases, the estimated health professional visit costs rose from a crude mean of $2174 (SE = 454) in the control phase to $3100 (SE = 524) in the intervention phase, resulting in an adjusted mean difference of $925 (95% CI: −488 to 2339). Hospital admission costs increased significantly from a crude mean of $20,481 (SE = 11,473) to $52,670 (SE = 18,549) in the intervention phase, resulting in an adjusted mean difference of $32,948 (95% CI: −10,888 to 76,784). This pattern of cost increase was similar for other services, including emergency admission and medication costs. However, informal care costs showed a decline from a crude mean of $25,067 (SE = 2945) in the control phase to $24,949 (SE = 3048) in the intervention phase, with an adjusted mean difference of $‐1659 (95% CI: −9868 to 6551). Overall, total costs exhibited a significant increase from a crude mean of $68,925 (SE = 12,959) in the control phase to $133,660 (SE = 21,188) in the intervention phase, resulting in an adjusted mean difference of $67,146 (95% CI: 16,957 to 117,334).

### 3.3 Sensitivity Analysis

The base case analysis results showed significantly higher costs for recipients during the intervention phase compared to controls, with crude and adjusted mean differences of $64,735 (95% CI: 13,167 to 116,302) and $67,146 (95% CI: 16,957 to 117,334), respectively. This cost difference remained significant when conducting a sensitivity analysis, where the crude and adjusted mean difference (CI) was $64,044 (95% CI: $12,477 to $115,612) and $ 66,456 (95% CI: $16,267 to $116,644), respectively.

## 4 Discussion

This study aimed to demonstrate the health outcomes and economic costs associated with the PITCH intervention, primarily designed to enhance the competency of HCWs in delivering care, which in turn was expected to improve clients' experiences, leading to better health outcomes (including QoL) and a reduction in the burden on family caregivers as secondary outcomes. Furthermore, the intervention was expected to reduce the economic burden associated with both informal and formal healthcare services required by clients [10]. The findings have shown that the intervention had no significant effect on client health‐related QoL outcomes or dyadic relations with their caregiver (even when client outcomes were reported by proxy). For the family caregiver, similar to the clients, no significant differences were observed between the control and intervention phases in their self‐reported QoL, caregiver burden or dyadic relations with their care recipient.

The non‐significant findings in QoL may be attributed to several factors, including the small sample size and the progressive nature of dementia with age, which contributes to a decline in QoL outcomes and an increase in resource usage [36]. While the primary outcome of the intervention was to improve HCWs' sense of competency in providing care, it was expected to enhance clients' experiences and lead to better health outcomes [10]. Despite the non‐significant findings in clients and caregivers' outcomes, the PITCH intervention effectively improves homecare workers' sense of competence, dementia attitudes and knowledge [37]. Therefore, given that many people with dementia will receive care at home from their caregivers and home care workers, training programmes like PITCH remain crucial in educating home care workers to achieve high‐quality care. However, future research is warranted to explore efficient and cost‐effective ways of delivering the intervention.

Baseline formal and informal care costs clarified key cost drivers and the economic burden of dementia. Hospital admissions accounted for 66% of formal healthcare costs, while informal care comprised 44% of total costs, underscoring the role of caregivers. Formal healthcare costs were higher in the intervention phase, with positive mean differences across service categories. Although informal care costs decreased during the intervention, total costs (formal plus informal) were significantly higher than in the control phase.

The PITCH intervention was costed under two scenarios: one where we expected the HCWs to be paid to attend the training and one where it was assumed that they would complete the training in their personal time, which reduced the cost significantly (from $908 to $218 per dyad). However, expecting HCWs to complete the training in their own time is likely unfeasible, whereas paying the staff would increase staff engagement and subsequently result in better client outcomes. It is unclear what the outcomes would have been if staff had been expected to attend the training in their personal time. Additionally, the full impact of switching from face‐to‐face delivery to online delivery could not have been explored. Although face‐to‐face delivery showed a significant difference in the primary outcome (i.e., HCWs' sense of self‐competence in providing care services to people living with dementia), while online training showed no significant difference compared to no training [37], how this translates to client outcomes remains unknown since we could not link dyad outcomes to HCWs' training. While online delivery seems to be cheaper than face‐to‐face delivery in Scenario 1, the costs were similar in Scenario 2, which were mainly driven by the cost of the facilitator to deliver the workshop, which can be reduced if more HCWs can be accommodated within one workshop.

## 5 Limitations

Our findings need to be interpreted carefully due to some study limitations, particularly related to the small sample. Given that the power calculation was done for the primary outcome (i.e., HCWs' sense of self‐competence), the economic analysis was underpowered, which is a common problem in clinical trials [38]. Despite efforts to analyses the health outcomes and costs during both intervention and control phases, linking dyad outcomes to HCWs' training was difficult. This was primarily due to the fact that some clients were seen by multiple HCWs, while HCWs themselves attended numerous clients. Additionally, the frequency and type of services provided by HCWs to individual clients remained unclear. Furthermore, the study's small sample size and the impact of COVID‐19 on resource utilisation and intervention implementation, including the shift from face‐to‐face to online delivery, limits the generalisability of the findings. Moreover, the study did not include estimates for the cost of backfilling staff while HCWs underwent training, which is essential for a comprehensive understanding of intervention costs. This study is also limited by its short time horizon, which may have overlooked long‐term costs and consequences, such as costs associated with clients entering residential aged care. Further research is also needed with regard to the most efficient way of delivering the intervention.

## 6 Conclusions

This paper reports the health economic component of the broader PITCH trial. The trial was designed primarily to assess whether the intervention improved HCWs' sense of competence in providing dementia care, while this economic analysis focused on costs and selected secondary outcomes for clients or caregivers, including QoL, caregiver burden and dyadic interaction. PITCH was associated with higher total costs during the intervention phase, despite reduced informal care costs, and there was no clear evidence of improvement in the secondary client or caregiver health and QoL outcomes. These findings should therefore not be interpreted as evidence regarding the primary effectiveness of the overall PITCH intervention. Rather, they highlight the uncertainty around costs and downstream client and caregiver outcomes, particularly given the small sample, short follow‐up, inability to link dyad outcomes directly to HCWs' training exposure, and incomplete modelling of important costs such as residential care entry and staff retention. Future research, interpreted alongside the main trial paper, should examine the most efficient and cost‐effective ways to deliver dementia training for HCWs, including the role of staff engagement and training delivery mode.

## Funding

This research was part of the National Health and Medical Research Council Project Grant. The funder has no role in the design of the study, the collection, analysis or interpretation of the data or writing the manuscript.

## Ethics Statement

This pragmatic stepped‐wedge cluster RCT received institutional approval from the Austin Health Human Research Ethics Committee (Austin Hospital) HREC/45314/Austin‐2018.

## Consent

Written informed consent was obtained from each participant prior to participating in the study.

## Conflicts of Interest

The authors declare no conflicts of interest.

## Supporting information


**Table S1:** Participant distribution across recruitment period.
**Table S2:** Overview of the PITCH intervention cost.
**Table S3:** Unit Cost for professional visit and health services.
**Table S4:** Medication unit cost.
**Table S5:** Caregiver work status.
**Table S6:** Consolidated Health Economic Evaluation Reporting Standards (CHEERS) 2022 checklist.

## Data Availability

All data and materials are available within the manuscript and Supporting Information resources. Further information may be available by request to the corresponding author.
