# Supplementary material for: Cost‐Consequence Analysis of a Dementia Specialist Training Program for Home Care Workers: Results From a Stepped‐Wedge Cluster‐Randomised Controlled Trial
Source: Australas J Ageing. 2026 Oct 5;45(4):e70239. doi: 10.1111/ajag.70239 (PMC13635955; doi:10.1111/ajag.70239)
Supplement: Supplementary file 1 [file AJAG-45-0-s001.docx]

**Supplementary file**

Table S1 Participant distribution across recruitment period

| group | Category | Visit | | |
| --- | --- | --- | --- | --- |
|  |  | Baseline | Visit 2 | Visit 3 |
| Client | Control | 60 | 10 | - |
|  | Intervention | - | 28 | 24 |
| Carer | Control | 60 | 9 | - |
|  | Intervention | - | 32 | 24 |

Table S2 Overview of the PITCH intervention cost

| Component | Measurement | Valuation | Cost Total | Online | F2f | Comments |
| --- | --- | --- | --- | --- | --- | --- |
| Workshops – trainer preparation time | 18 hours | $62/h | $1,116 | $744 | $372 | Assume 1h preparation for 6 F2F clusters + 12 online =18h  $47.70/h +30% on-cost=$62 (rate of a project officer) |
| Workshops – trainer workshop delivery time | 114 hours | $62/h | $7,068 | $4,464 | $2,604 | 6 F2F workshops * 7 hours = 42 hours  12 online workshops * 6 hours = 72 hours  47.70/h +30% on-cost=$62 (rate of a project officer) |
| Workshops – HCW time | 1,163 hours (178 HCW) | $32.5 | $37,798 | $16,185 | $21,613 | 178 HCW who completed the training (partially or full)  F2F: 95 HCW * 7 hours =665h  Online: 83 HCW * 6 hours=498h  25/h +30% on-cost=$32.5 (HCW) |
| Catering for training session | 12 workshops | $30 | $360 |  | $360 | $30 per workshop (6 x 2 F2F workshops) |
| Materials | 1 workbook for 178 HCW | $10.6 | $1,887 | $880 | $1,007 | 53pages * $0.20 per page=$10.6 per workbook  83 HCWs (online) + 95 HCSs (F2F) = 178 |
| Travel time – Trainer | 14 hours | $62/h | $868 |  | $868 | Clusters A,B,C travel to NSW (2 trips x 6 hours)  Clusters E,G,F in VIC (assume 2 trips x 1 hour) |
| Travel cost – Trainer | 2 trips  2 trips | $500 (A,B,C)  $36 | $1000 + $72  =$1072 |  | $1,072 | Clusters A,B,C travel to NSW (2 trips x $500=$1000)  Clusters E,G,F in VIC (assume 50km trip * $0.72=36 * 2 trips=$72)  ATO rates: 72 cents per kilometre for 2020–21 |
| Travel time – HCWs | 47.5 hours (95 HCW) | $32.5 | $1,544 |  | $1,544 | Assume 30 mins trip for travel (round trip)  F2F: 95 HCW * 0.5h = 47.5 hours |
| Travel cost – HCWs | 2850 km | $0.72 | $2,052 |  | $2,052 | Assume 30km trip (round trip)  ATO rates: 72 cents per kilometre for 2020–21  F2F: 95 HCW * 30 = 2850 km |
| Organizing workshop | 18 hours | $39 | $702 | $468 | $234 | Assume 0.5h preparation for 6 x2 F2F clusters = 6h  Assume 0.5h preparation for 12 x2 online clusters = 12h  $30/h +30% on-cost (rate of a project officer) |
| Scenario 1: HCWs are paid to do the training. Based on ACTUAL participation rates (178 HCW who completed the training (partially or full)) | | | | | | |
| TOTAL cost |  |  | $54,467 | $21,997 | $31,725 | TOTAL: 178 HCW, 18 providers  Online: 83 HCWs, 12 providers  F2F: 95 HCWs, 6 providers |
| *Total cost per HCW* |  |  | *$306* | *$265* | *$333* |  |
| *Total cost per provider* |  |  | $3,026 | $1,833 | $5,288 |  |
| Scenario 2: The service provider expects the HCWs to do the training in their own time | | | | | | |
| TOTAL cost |  |  | $13,073 | $6,556 | $6,517 |  |
| *Total cost per HCW* |  |  | $73 | $79 | $69 |  |
| *Total cost per provider* |  |  | $726 | $546 | $1,086 |  |

Table S3 Unit Cost for professional visit and health services

| Health services | Unit | Total services | Total benefit | Unit cost | Source |
| --- | --- | --- | --- | --- | --- |
| Hospital admission | Number (>24 hours) | 6141848.0 | 32765824485.0 | 5334.8 | [NHCDC Public Sector, Round 24](https://www.ihpa.gov.au/publications/national-hospital-cost-data-collection-public-sector-round-24-financial-year-2019-20) |
| Nights in ward | The same cost applied | | | |  |
| Geriatric |  |  |  |  |  |
| Psychiatry |  |  |  |  |  |
| Internal medicine |  |  |  |  |  |
| Surgery |  |  |  |  |  |
| Neurology |  |  |  |  |  |
| General ward |  |  |  |  |  |
| Other (please specify) |  |  |  |  |  |
| Professional Visit | Unit |  |  |  |  |
| General Practitioner (MBS item 3, 23, 36, 44, 91790, 91800, 91801, 91802,  91795, 91809, 91810, 91811) | Visit | 127048141.0 | 5602648283.0 | 44.1 | MBS January 2020 |
| Geriatrician (MBS item 141, 143, 145, 147, 92624, 92629) | Visit | 86662.0 | 32593209.0 | 376.1 |  |
| Neurologist (MBS item 6007, 6009, 6011, 6013, 6015) | Visit | 219798.0 | 20244582.0 | 92.1 |  |
| Psychiatrist (MBS item 296, 297, 299, 291, 293, 359, 361) | Visit | 183589.0 | 49188379.0 | 267.9 |  |
| Physiotherapist (MBS item 10960, 81335 (aboriginal) | Visit | 2691862.0 | 146434676.0 | 54.4 |  |
| Occupational therapist (MBS item 10985, 80125, 80130, 80135, 80140, 80145, 81330, 80126, 80136,  91172, 91173, 91185, 91186) | Visit | 64054.0 | 8034059.0 | 125.4 |  |
| Social worker (MBS item 80150, 80151, 80155, 80160, 80161, 80165, 91175, 91176, 91187, 91188) | Visit | 388452.0 | 31453975.0 | 81.0 |  |
| Psychologist (MBS item 10968, 80000, 80005, 80010, 80015, 80100, 80105, 80110, 80115, 80001,  80011, 80101, 80111, 91166, 91167, 91169, 91170, 91181, 91182, 91183, 91184) | Visit | 5540288.0 | 600871650.0 | 108.5 |  |
| Urologist (MBS items 37227) | Visit | 70.0 | 28815.0 | 411.6 |  |
| Podiatrist (MBS item 10962, 81340) | Visit | 3463744.0 | 187087661.0 | 54.0 |  |
| Surgeon (MBS items 10, 105, 110, 132, 133) | Visit | 9993491.0 | 872356905.0 | 87.3 |  |
| Vascular surgeon (MBS items 36506, 36509) | Visit | 81.0 | 40805.0 | 503.8 |  |
| Nurse (MBS item 82200, 82205, 82210, 82215, 91178, 91179, 91180, 91189, 91190, 91191, 91192, 91193) | Visit | 668275.0 | 20455364.0 | 30.6 |  |
| Pain management specialist (MBS item 2801, 2806, 2814, 2824, 2832, 2840) | Visit | 113956.0 | 10439878.0 | 91.6 |  |
| Cardiologist (MBS item 6080, 6081) | Visit | 2012.0 | 89389.0 | 44.4 |  |
| Heart specialist | Visit |  |  |  |  |
| Optometrist (MBS item 10945, 10946, 10947, 10948) | Visit | 683.0 | 31356.0 | 45.9 |  |
| Ophthalmologist (MBS item 12325, 12326 ) | Visit | 3033.0 | 130984.0 | 43.2 |  |
| Eye specialist | Visit |  |  |  |  |
| Exercise physiologist | Visit | 307784.0 | 16769292.0 | 54.5 |  |
| Exercise fitness | Visit |  |  |  |  |
| Kidney specialist (MBS item 13104, 13105) | Visit | 25553.0 | 7812359.0 | 305.7 |  |
| Dentist (MBS item 75800) | Visit | 1351.0 | 96792.0 | 71.6 |  |
| Chiropractor (MBS item 10964, 81345) | Visit | 500555.0 | 26965527.0 | 53.9 |  |
| Massage therapist | Visit |  |  |  |  |
| Volunteers to mow the grass | per hour |  |  | 62.0 | <https://www.promptcare.com.au/service.strc.fees.php> |
| Lite and easy meals | per hour |  |  | 62.0 |  |
| Hair dresser | per hour |  |  | 62.0 |  |
| Hospital services | Unit |  |  |  |  |
| Emergency | Number | 8172976.0 | 6332992326.0 | 774.9 | [NHCDC Public Sector, Round 24](https://www.ihpa.gov.au/publications/national-hospital-cost-data-collection-public-sector-round-24-financial-year-2019-20) |
| District nurse (MBS items 82205, 82210, 82215) | Number | 588546.0 | 18915449.0 | 32.1 | MBS January 2020 |
| Home help/healthcare assistant | per hour |  |  | 48.1 | <https://www.promptcare.com.au/service.strc.fees.php> |
| Meals on Wheels | per hour |  |  | 62.0 |  |
| Day care | per hour |  |  | 62.0 |  |
| Transportation (care related) | per hour |  |  | 62.0 |  |

Table S4 Medication Unit Cost

| Generic name of medicine | Quantity/schedule | DPMQ | Max patient charge | General patient | Concessional patient | Australian Government |
| --- | --- | --- | --- | --- | --- | --- |
| Movicol | 30 sachet, 1/daily | 19.05 | 24.9 | 41 | 6.6 | 12.45 |
| Allopurinol | 200 tab, 100mg/daily | 16.34 | 22.19 | 41 | 6.6 | 9.74 |
| Allopurinol | 60 tab, 300ml (300mg)/30 days | 15 | 20.85 | 41 | 6.6 | 8.4 |
| Amitriptyline-AF | 50 tab, 10mg/daily | 13.99 | 19.84 | 41 | 6.6 | 7.39 |
| Amlodipine | 30tab, 5mg/daily | 12.37 | 18.22 | 41 | 6.6 | 5.77 |
| Apixaban | 30 tab, 2.5/BID | 52.4 | 41 | 41 | 6.6 | 45.8 |
| Aricept (donepezil) | 10mg/daily | 21.54 | 27.39 | 41 | 6.6 | 14.94 |
| Asmol | 2puffs/TID | 19.87 | 25.72 | 41 | 6.6 | 13.27 |
| Aspirin | 90 tab, 100mg/daily | 19.52 | 6.6 | 41 | 6.6 | 12.92 |
| Aspirin EC | 84 tab, 100mg/daily | 17.82 | 6.6 | 41 | 6.6 | 11.22 |
| Atenolol | 12.5mg/daily | 12.62 | 18.47 | 41 | 6.6 | 6.02 |
| Atenolol | 30 tab, 50mg X 1/2 tab/daily | 12.62 | 18.47 | 41 | 6.6 | 6.02 |
| Atorvastatin (Lipitor) | 30 tab, 10mg/daily | 13.01 | 18.86 | 41 | 6.6 | 6.41 |
| Atorvastatin | 30 tab, 20mg/daily | 13.74 | 19.59 | 41 | 6.6 | 7.14 |
| Atorvastatin | 30 tab, 40mg/daily | 14.65 | 20.5 | 41 | 6.6 | 8.05 |
| Attacand | 30 tab, 8mg | 12.87 | 32.19 | 41 | 6.6 | 6.27 |
| Aucloxacillin /Dicloxacillin/ | 24 cap, Dicloxacillin 500 mg | 19.58 | 25.43 | 41 | 6.6 | 12.98 |
| Augmentin duo | 10 tab, 875/125 | 14.25 | 20.1 | 41 | 6.6 | 7.65 |
| Mirtazapine (Axit) | 30 tab, 15mg, 0.5/daily | 13.88 | 19.73 | 41 | 6.6 | 7.28 |
| Baclofen | 25mg x 2/BD | 33.29 | 39.14 | 41 | 6.6 | 26.69 |
| Caltrate | 120 tab, 600mg/daily | 17.05 | 6.6 | 41 | 6.6 | 10.45 |
| Cavstat (rosuvastatin) | 30 tab, 20mg/daily | 19.34 | 25.19 | 41 | 6.6 | 12.74 |
| Celecoxib | 60 tab, 100mg/daily | 16.43 | 22.28 | 41 | 6.6 | 9.83 |
| Chlorambucil | 25 tab, 2.25mg/daily | 133.63 | 41 | 41 | 6.6 | 127.03 |
| Claratyne (loratidine) | 30 tab, 10mg/daily | 30.88 | 6.6 | 41 | 6.6 | 24.28 |
| Coloxyl | 100 tab | 18.34 | 6.6 | 41 | 6.6 | 11.74 |
| Coloxyl and Senna | 90 tab, 50 mg/BD | 17.32 | 6.6 | 41 | 6.6 | 10.72 |
| Coversyl | 30 tab, 2mg/daily | 13.02 | 18.87 | 41 | 6.6 | 6.42 |
| Coversyl | 30 tab, 5mg/daily | 14.34 | 27.2 | 41 | 6.6 | 7.74 |
| Desvenlafaxine MR | 28 tab, 100mg/daily | 23.97 | 29.82 | 41 | 6.6 | 17.37 |
| Desvenlafaxine MR | 28 tab, 50mg/daily | 21.65 | 27.5 | 41 | 6.6 | 15.05 |
| Diltiazem | 30 tab, 360mg/daily | 23.39 | 29.24 | 41 | 6.6 | 16.79 |
| Ditropan /oxybutynin/ | 100 tab, 5mg/daily | 15.29 | 21.14 | 41 | 6.6 | 8.69 |
| Donepezil | 28 tab, 10mg/daily | 21.54 | 27.39 | 41 | 6.6 | 14.94 |
| Doneprezil | 28 tab, 5mg x 1/2 /daily | 21.54 | 27.39 | 41 | 6.6 | 14.94 |
| Duro K | 200 tab, 600mg/daily | 21.16 | 27.01 | 41 | 6.6 | 14.56 |
| Dutasteride/tams | 500mcg-400mcg/daily | 32.36 | 6.6 | 41 | 6.6 | 25.76 |
| Ebixa | 56 tab, 10mg/daily | 41.68 | 41 | 41 | 6.6 | 35.08 |
| Eliquis | 60 tab, 5mg/BID | 93.31 | 41 | 41 | 6.6 | 86.71 |
| Esomeprazole | 30 cap/tab, 20mg/daily | 17.79 | 23.64 | 41 | 6.6 | 11.19 |
| Ezsimva | 30 tab, 10/80mg/daily | 37.24 | 41 | 41 | 6.6 | 30.64 |
| Fentanyl patch | 5 patch, 25mcg/q3d | 25.35 | 31.2 | 41 | 6.6 | 18.75 |
| Ferro F | 60 tab, 350mcg/BD | 19.17 | 6.6 | 41 | 6.6 | 12.57 |
| Frusemide | 100 tab, 20mg/daily | 13.16 | 19.01 | 41 | 6.6 | 6.56 |
| Frusemide | 100 tab, 40mg/daily | 13.16 | 19.01 | 41 | 6.6 | 6.56 |
| Gabapentin | 100tab, 600mg/BID | 40.7 | 6.6 | 41 | 6.6 | 34.1 |
| Galvus (Vildagliptin) | 60 tab, 50mg/daily | 60 | 41 | 41 | 6.6 | 53.4 |
| GliclazideMR | 60 tab, 60mg/daily | 18.72 | 24.57 | 41 | 6.6 | 12.12 |
| Glyade MR/Gliclazide/ | 100tab, 30mg/daily | 17.51 | 23.36 | 41 | 6.6 | 10.91 |
| Hipprex | 100 tab, 1g | 39.71 | 41 | 41 | 6.6 | 33.11 |
| Indosyl mono/Perindopril/ | 30 tab, 4mg X 1.5/daily | 14.34 | 20.19 | 41 | 6.6 | 7.74 |
| Isosorbide mononitrate | 30 tab, 60mg/daily | 15.16 | 21.01 | 41 | 6.6 | 8.56 |
| Januvia/sitagliptin/ | 28 tab, 50mg/daily | 52.24 | 41 | 41 | 6.6 | 45.64 |
| Jardiance /Empagliflozin/ | 30 tab, 10mg/daily | 62.03 | 41 | 41 | 6.6 | 55.43 |
| Kombiglyze /Saxagliptin with metformin/ | 28 tab, 5mg/1000mg/daily | 58.27 | 41 | 41 | 6.6 | 51.67 |
| Lantus pen (insulin) /Insulin glargine/ | 100 units, 14 units | 181.94 | 41 | 41 | 6.6 | 175.34 |
| Latanaprost eye drops | 1 drop/daily | 17.31 | 23.16 | 41 | 6.6 | 10.71 |
| Senna | 100 tab, tab/BID | 16.85 | 6.6 | 41 | 6.6 | 10.25 |
| Zanipid (Lercanidipine) | 28 tab, 20mg/daily | 14.97 | 20.82 | 41 | 6.6 | 8.37 |
| Levothyroxine | 200 tab, 100mcg/daily | 26.75 | 32.6 | 41 | 6.6 | 20.15 |
| Lisinopril | 30 tab, 10mg/daily | 14.72 | 20.57 | 41 | 6.6 | 8.12 |
| Lyrica/PREGABALIN/ | 56 cap, 75mg/BID | 28.32 | 34.17 | 41 | 6.6 | 21.72 |
| Mag-sup | 50 tab, 500 mg/daily | 17.5 | 6.6 | 41 | 6.6 | 10.9 |
| Marevan /warfarin/ | 50 tab, 3mg/4mg alternate days/daily | 16.42 | 22.27 | 41 | 6.6 | 9.82 |
| Memantine GH | 28 tab, 20mg/daily | 41.68 | 41 | 41 | 6.6 | 35.08 |
| Memantine hydrochloride | 5mg/daily | 41.68 | 41 | 41 | 6.6 | 35.08 |
| Metformin | 60 tab, 1000mg/daily | 15.79 | 21.64 | 41 | 6.6 | 9.19 |
| Metformin | 120 tab, 500mg/daily | 15.79 | 21.64 | 41 | 6.6 | 9.19 |
| Metoclopramide | 25 tab, 10mg/daily | 12.61 | 18.46 | 41 | 6.6 | 6.01 |
| Metoprolol | 60 tab, 100mg 1/2 tab mane and x 1 tab nocte/daily | 13.91 | 19.76 | 41 | 6.6 | 7.31 |
| Minax /metoprolol/ | 100 tab, 50mg 1/2 tab /BD | 13.38 | 19.23 | 41 | 6.6 | 6.78 |
| Mirtanza | 30 tab, 15mg/daily | 13.88 | 19.73 | 41 | 6.6 | 7.28 |
| Monodur | 30 tab, 120mg/daily | 18.8 | 24.65 | 41 | 6.6 | 12.2 |
| Nicorandil | 60 tab, 10mg/BID | 21.71 | 27.56 | 41 | 6.6 | 15.11 |
| Osteomol | 96 tab, 665/BID | 18.95 | 6.6 | 41 | 6.6 | 12.35 |
| Sinemet(levodopa&carbidopa) | 100 tab, 250/25mg/TDS 30 | 45.65 | 41 | 41 | 6.6 | 39.05 |
| Oxazepam | 25 tab, 30mg/daily | 12.56 | 18.41 | 41 | 6.6 | 5.96 |
| PANADEINE FORTE (Paracetamol &Codein) | 20 tab, 500mg/daily | 12.83 | 20.78 | 41 | 6.6 | 6.23 |
| Panadol /Paracetamol/ | 100 tab, 500mg/BID | 13.13 | 18.98 | 41 | 6.6 | 6.53 |
| Pantoprazole | 20 tab, 20 mg | 12.73 | 18.58 | 41 | 6.6 | 6.13 |
| Pantoprazole (somac) | 30 tab, 40 mg/daily | 14.03 | 19.88 | 41 | 6.6 | 7.43 |
| Peridopril | 30 tab, 10mg/daily | 15.86 | 21.71 | 41 | 6.6 | 9.26 |
| Perindopril | 30 tab, 2mg/daily | 13.02 | 18.87 | 41 | 6.6 | 6.42 |
| Perindopril | 30 tab, 5mg/daily | 14.34 | 20.19 | 41 | 6.6 | 7.74 |
| Perindopril | 30 tab, 8mg/daily | 15.86 | 21.71 | 41 | 6.6 | 9.26 |
| Plavix | 28 tab, 65mg/daily | 15.57 | 6.6 | 41 | 6.6 | 8.97 |
| Pravistatin | 30 tab, 40mg/daily | 15.28 | 21.13 | 41 | 6.6 | 8.68 |
| Pregabalin | 56 cap, 150mg/BID | 37.31 | 41 | 41 | 6.6 | 30.71 |
| Prexum /perindopril/ | 2.5mg/daily | 13.02 | 18.87 | 41 | 6.6 | 6.42 |
| Prolia /DENOSUMAB/ | 1 injec, 60mg/every six month | 271.88 | 41 | 41 | 6.6 | 265.28 |
| syquet (Quetiapine) | 60 tab, 25mg(0.5)/daily | 17.32 | 23.17 | 41 | 6.6 | 10.72 |
| Quetiapine | 90 tab, 100mg/daily | 28 | 33.85 | 41 | 6.6 | 21.4 |
| Risperdal/Risperidone/ | 60 tab, 0.5mg(2)/daily | 18.61 | 24.46 | 41 | 6.6 | 12.01 |
| Rosuvastatin | 30 tab, 10mg/daily | 17.01 | 22.86 | 41 | 6.6 | 10.41 |
| Rosuvastatin | 5mg/daily | 15.24 | 21.09 | 41 | 6.6 | 8.64 |
| SeeBreeze Inhaler | 30 cap, 50mcg/daily | 59.81 | 41 | 41 | 6.6 | 53.21 |
| Seretide/Fluticasone propionate with salmeterol/ | BID | 48.64 | 41 | 41 | 6.6 | 42.04 |
| Sertraline | 30 tab, 100mg/daily | 13.17 | 19.02 | 41 | 6.6 | 6.57 |
| Sigmaxin /digoxin/ | 200 tab, 62.5mcg/daily | 14.96 | 20.81 | 41 | 6.6 | 8.36 |
| Simvastatin | 30 tab, 20mg/daily | 13.1 | 18.95 | 41 | 6.6 | 6.5 |
| Slucloxacillin | 5 vial, 1gm/QID | 20.06 | 25.91 | 41 | 6.6 | 13.46 |
| Sodium Valprate | Syrup, 200gms(5mls)/BD | 39.45 | 41 | 41 | 6.6 | 32.85 |
| Sotalol | 60 tab, 80mg (1/2 tablet)/BID | 14.69 | 20.54 | 41 | 6.6 | 8.09 |
| Spiriva inhaler | 1 (60 actuations) 2.5mcg x 2 puffs/daily | 52.8 | 41 | 41 | 6.6 | 46.2 |
| Spirolactone | 100 tab, 25mg /daily | 16.65 | 22.35 | 41 | 6.6 | 10.05 |
| Systane eye drops | 1 drop/daily | 14.44 | 20.29 | 41 | 6.6 | 7.84 |
| Tegretol/Carbamazepine/ | 100 tab, 200mg/daily | 31.13 | 36.98 | 41 | 6.6 | 24.53 |
| Tegretol | 200 tab, 400mg/daily | 49.59 | 41 | 41 | 6.6 | 42.99 |
| Telmisartan | 28 tab, 40mg/daily | 13.25 | 19.1 | 41 | 6.6 | 6.65 |
| Temaze | 25 tab, 10mg/daily | 13.65 | 19.5 | 41 | 6.6 | 7.05 |
| Thyroxine | 200 tab, 100mcg/daily | 26.75 | 32.6 | 41 | 6.6 | 20.15 |
| Vaclovir | 500mg/daily | 25.74 | 31.59 | 41 | 6.6 | 19.14 |
| Venlafaxine | 28 tab, 75mg/daily | 15.08 | 20.93 | 41 | 6.6 | 8.48 |
| Vit B12 | 1 inject, 1000mcg/daily | 15.75 | 6.6 | 41 | 6.6 | 9.15 |
| Xarelto | 28 tab, 15mg /daily | 87.56 | 41 | 41 | 6.6 | 80.96 |
| Xarelto | 28 tab, 20mg/daily | 87.56 | 41 | 41 | 6.6 | 80.96 |

Table S5 Caregiver work status

| Caregiver work status | | Baseline N (%) |
| --- | --- | --- |
| Working status | Yes | 9 (18) |
|  | No | 42 (82) |
| Why stop working (n=42) | Retirement (reached or early not disease-related) | 24 (57) |
|  | To care client | 10 (24) |
|  | Others | 4 (110) |
|  | Own health problems | 1 (2) |
|  | Never worked professionally | 1 (2) |
| Income source (n=42) | Pension | 21 (55) |
|  | Savings | 10 (26) |
|  | Others | 7 (19) |
| Productivity loss /missed work (n=9) | Whole day | 8 (89) |
|  | Part of a day | 1 (11) |

Table S6 Consolidated Health Economic Evaluation Reporting Standards (CHEERS) 2022 Checklist

| Topic | No. | Item | Location where item is reported |
| --- | --- | --- | --- |
| Title |  |  |  |
|  | 1 | Cost-consequence analysis of a dementia specialist training program for home care workers: Results from a stepped-wedge cluster-randomized controlled trial. | Title, Title Page 1 |
| Abstract |  |  |  |
|  | 2 | Provide a structured summary that highlights context, key methods, results, and alternative analyses. | Abstract, Page 2 |
| Introduction |  |  |  |
| Background and objectives | 3 | Give the context for the study, the study question, and its practical relevance for decision making in policy or practice. | Introduction, page 3 |
| Methods |  |  |  |
| Health economic analysis plan | 4 | Indicate whether a health economic analysis plan was developed and where available. | Methods, page 7 |
| Study population | 5 | Describe characteristics of the study population (such as age range, demographics, socioeconomic, or clinical characteristics). | Methods, page 4 |
| Setting and location | 6 | Provide relevant contextual information that may influence findings. | Methods, page 4 |
| Comparators | 7 | Describe the interventions or strategies being compared and why chosen. | Methods, page 4 |
| Perspective | 8 | State the perspective(s) adopted by the study and why chosen. | Methods, page 6 |
| Time horizon | 9 | State the time horizon for the study and why appropriate. | Methods, page 6 |
| Discount rate | 10 | Report the discount rate(s) and reason chosen. | N/A (Short time horizon) |
| Selection of outcomes | 11 | Describe what outcomes were used as the measure(s) of benefit(s) and harm(s). | Methods, page 4 |
| Measurement of outcomes | 12 | Describe how outcomes used to capture benefit(s) and harm(s) were measured. | Methods, page 4 to 5 |
| Valuation of outcomes | 13 | Describe the population and methods used to measure and value outcomes. | Methods, page 6 to 7 |
| Measurement and valuation of resources and costs | 14 | Describe how costs were valued. | Methods, page 6 and 7 |
| Currency, price date, and conversion | 15 | Report the dates of the estimated resource quantities and unit costs, plus the currency and year of conversion. | Methods, page 7 |
| Rationale and description of model | 16 | If modelling is used, describe in detail and why used. Report if the model is publicly available and where it can be accessed. | N/A |
| Analytics and assumptions | 17 | Describe any methods for analysing or statistically transforming data, any extrapolation methods, and approaches for validating any model used. | N/A |
| Characterising heterogeneity | 18 | Describe any methods used for estimating how the results of the study vary for subgroups. | N/A |
| Characterising distributional effects | 19 | Describe how impacts are distributed across different individuals or adjustments made to reflect priority populations. | N/A |
| Characterising uncertainty | 20 | Describe methods to characterise any sources of uncertainty in the analysis. | N/A |
| Approach to engagement with patients and others affected by the study | 21 | Describe any approaches to engage patients or service recipients, the general public, communities, or stakeholders (such as clinicians or payers) in the design of the study. | N/A |
| Results |  |  |  |
| Study parameters | 22 | Report all analytic inputs (such as values, ranges, references) including uncertainty or distributional assumptions. | N/A |
| Summary of main results | 23 | Report the mean values for the main categories of costs and outcomes of interest and summarise them in the most appropriate overall measure. | Results, page 7 to 9 |
| Effect of uncertainty | 24 | Describe how uncertainty about analytic judgments, inputs, or projections affect findings. Report the effect of choice of discount rate and time horizon, if applicable. | N/A |
| Effect of engagement with patients and others affected by the study | 25 | Report on any difference patient/service recipient, general public, community, or stakeholder involvement made to the approach or findings of the study | N/A |
| Discussion |  |  |  |
| Study findings, limitations, generalisability, and current knowledge | 26 | Report key findings, limitations, ethical or equity considerations not captured, and how these could affect patients, policy, or practice. | Discussion, page 10 to 12 |
| Other relevant information |  |  |  |
| Source of funding | 27 | Describe how the study was funded and any role of the funder in the identification, design, conduct, and reporting of the analysis | Title page |
| Conflicts of interest | 28 | Report authors conflicts of interest according to journal or International Committee of Medical Journal Editors requirements. | Title page |

***Source:*** Husereau D, Drummond M, Augustovski F, et al. Consolidated Health Economic Evaluation Reporting Standards 2022 (CHEERS 2022) Explanation and Elaboration: A Report of the ISPOR CHEERS II Good Practices Task Force. Value Health 2022;25. <doi:10.1016/j.jval.2021.10.008>
